# Androgen Receptor Promotes Gastric Carcinogenesis via Upregulating Cell Cycle-Related Kinase Expression

**DOI:** 10.7150/jca.34430

**Published:** 2019-07-10

**Authors:** Ren Wang, Xiao-yi Xu, Hong Zhu, Xiong Liang, Xue Li, Ming-xu Jia, Qing-hua Wang, Hui-yun Wang, Xiao-xing Li, Gui-jun Zhao

**Affiliations:** 1 State Key Laboratory of Oncology in South China, Collaborative Innovation Center for Cancer Medicine, Sun Yat-Sen University Cancer Center, Guangzhou, Guangdong, China; 2 Endoscopy Center, Inner Mongolia key laboratory of endoscopic digestive diseases, Inner Mongolia People's Hospital, Hohhot, Inner Mongolia Autonomous Region, China

**Keywords:** AR, CCRK, gastric cancer, prognosis

## Abstract

Gastric cancer (GC) is a leading global health problem as it is the fifth most common cancer type and the third most common cause of cancer-related deaths worldwide. In most areas of the world, the incidence rate of GC is 1.5- to 3-fold higher in males than in females. The androgen receptor (AR) is an independent adverse prognostic factor in patients with GC. However, the mechanism by which AR regulates the progression of GC remains unclear. In this study, we found that AR expression was upregulated in 6/8 GC cell lines, and this expression was higher than that in immortalized gastric cells. AR expression was also higher in GC tissues than in adjacent tissues. Moreover, the ectopic expression of AR promoted the colony formation ability, migration and invasion of GC cells. In contrast, AR knockdown had the opposite effects on GC cell lines. Remarkably, we found that AR regulated cell cycle-related kinase (CCRK) expression through transcriptional mechanisms. The AR-CCRK axis promoted GC development through the phosphorylation of GSK3β and β-catenin. Furthermore, TCGA data revealed that high expression of AR or CCRK was related to poor prognosis in GC patients. The prognosis was significantly worse in patients with concurrent high AR and CCRK expression than in patients with low AR and CCRK expression. In conclusion, our study demonstrated that AR and CCRK acted as oncogenes in GC progression. However, their clinical roles require further exploration.

## Introduction

Gastric cancer (GC) remains a global health problem as it is the fifth most common cancer and the third most common cause of cancer-related deaths worldwide. Globally, the incidence of GC was 1,000,000 cases in 2018 1. It is estimated that the total number of GC deaths in 2018 was 783,000, accounting for 8.3% of cancer-related death 1. The incidence of GC in males is approximately 1.5-3 times that in females 2. Some data suggest that estrogen provides potential protection in women. Hormone replacement therapy reduces the risk of GC by more than 50% 3. Tamoxifen is a selective estrogen receptor modulator, and breast cancer patients treated with tamoxifen showed an increased risk of developing GC 4. Animal studies suggested that, compared with untreated male rats, castrated or estrogen-treated male rats displayed a lower incidence of GC 5. Ulanova M.* et al.* demonstrated that sex could affect stress-induced stomach cancer progression in adult rats through oxygenation regulation 6. Many studies have demonstrated the beneficial effects of female estrogen against GC, but male-related factors that promote GC have seldom been researched. Therefore, we aimed to study the role of AR in the progression of GC.

The androgen/AR signaling pathway is involved in the development of male-specific phenotypes during embryonic development, spermatogenesis, sexual behavior and adult reproduction 7. AR is a member of the nuclear steroid receptor superfamily of transcription factors, which includes three main functional domains: the N-terminal domain, the DNA binding domain and the C-terminal ligand binding domain 8. Many studies have examined AR in different cancers. For example, in prostate and pancreatic cancer cells, AR increased cell proliferation, invasion and apoptosis, but these effects were inhibited by miRNA-488 or miRNA-101 9-11. Increasing the interaction between AR and cytoskeletal protein filamin A was found to be the mechanism of how AR promoted cell migration 12. Furthermore, MA W.L. *et al.* found that AR could indirectly suppress NFκB to inhibit invasion in HCC 13. Studies have also shown that AR promoted the proliferation of ER-/HER2+ breast tumor cells and activated the Wnt and HER2 signaling pathways by promoting the expression of HER3 and WNT7B 14. AR also enhanced MYC transcriptional activity by phosphorylating MAD1 and promoting activation of the HER2/HER3 signaling pathway, leading to increased levels of MYC/MAX heterodimers that ultimately promoted breast cancer growth 15. However, AR has not been further characterized biochemically, and its function remains unknown in GC.

The aim of this study is to determine the effect of AR on the progression of GC and the mechanism underlying these effects, which will provide novel ideas for the treatment of GC.

## Materials and Methods

### Cell lines and cell culture

In our research, 7 human GC cell lines (AGS, SGC7901, MKN45, MKN28, HGC27, BGC823, and MGC803) and 1 normal immortalized gastric mucosal cell line GES-1 were used for the assays. AGS and MGC803 cells were obtained from the ATCC. MKN45, MKN28, HGC27, BGC-823 and SGC7901 cells were purchased from the Cancer Hospital of the Chinese Academy of Medical Sciences (Beijing, China). MKN45, MKN28, HGC27 and SGC7901 cells were cultured in DMEM (Gibco, USA) with 10% FBS (BI, USA). AGS, MGC-803, BGC-823 and SGC-7901 cells were cultured in RPMI-1640 medium (Gibco, USA) supplemented with 10% FBS (BI, USA). Cells were cultured with 100 μg/mL penicillin and 100 μg/mL streptomycin in a humid atmosphere containing 5% CO_2_ at 37 °C until they reached 80% confluency. The cells were then digested with 0.25% trypsin and inoculated into a new flat.

### Clinical samples

Four pairs of clinical tissue samples from primary tumors and adjacent nontumorous sites were collected from the Inner Mongolia People's Hospital (Inner Mongolia, China). The project was approved by the Ethics Committee of Clinical Research of the Inner Mongolia People's Hospital.

### Gene enrichment analysis (GSEA)

We downloaded the gene expression data of 440 clinical gastric adenocarcinoma samples (TCGA, Pan-Cancer Atlas) from the cBioPortal database. Then, we used Rstudio software to develop a program to simulate the high or low expression of AR by selecting 30 samples randomly from 440 clinical samples that had the 25% highest or 25% lowest AR expression values and then used this gene set for GSEA. GSEA software analysis included the following four important factors: enrichment score (ES), normalized enrichment score (NES), false discovery rate (FDR) and P value.

### Semiquantitative RT-PCR and real-time quantitative PCR

Total RNA was extracted from GC cells and tumor tissues using triazole reagent (Ambion, USA, 15596018). cDNA was synthesized using a GoScript reverse transcription kit (Promega, USA, A5001). The relative expression of AR and CCRK was detected by semiquantitative RT-PCR and real-time quantitative PCR. Primers: AR RT-sense: 5'-AAGCCAGAGCTGTGCAGATGA-3'; AR-RT-anti-sense: 5'-TGTCCTGCAGCCACTGGTTC-3'; CCRK-RT-sense: 5'-AGACTGGCGAGATAGTTGCC-3'; CCRK-RT-anti-sense: 5'-GTGGGAACACAGCCTTCAGT-3'.

### Transwell migration and invasion assay

GC cells were digested to produce a single-cell suspension, which was then diluted to a concentration of 1X10^5^ cells/mL with serum-free medium. Then, 200-μl cell suspensions were placed in a transwell upper chamber (for the migration assay) or a Matrigel invasion chamber (for the invasion assay). The lower chamber contained 10% fetal bovine serum culture medium and was maintained at 37 °C with 5% CO_2_ for cultivation for six to eight hours (for the migration assay) or for 18 to 24 hours (for the invasive assay). Adherent cells were fixed with 4% paraformaldehyde and stained with crystal violet. The cells were counted with an optical microscope.

### Colony formation experiment

AR cells were digested with 0.25% trypsin, and the cell suspension was diluted to 1X10^4^ cells/mL. Then, 1000 cells were plated on a 6-well plate. Cell culture conditions were 37 °C for 7-14 days. The colony number was counted after staining with crystal violet.

### Chromatin immunoprecipitation (ChIP) and PCR

ChIP analysis was performed using anti-AR antibody (Abcam: #5153) and IgG (Abcam: #3900) according to the manufacturer's protocol. PCR was performed using the following DNA promoter-specific primers: CCRK-RT-promoter-sense: 5'-CGCAACGGCCCAAAGTAG-3'; CCRK-RT-promoter-anti-sense: 5'-CGCAACGGCCCAAAGTAG-3'.

### *In vivo* tumorigenicity

CCRK was transformed with or without vector into AGS cells. BGC823 cells were transfected with shCCRK, shVector or no vector. The cell concentration was adjusted to 1 x 10^7^ cells/mL. We assigned five 6-week-old BALB/c nude mice to each group and then injected 200 μL of the cell suspension into their right dorsal sides. We collected data, including mouse weight and tumor volume, every week. Following observation for 6 weeks, the nude mice were sacrificed; the stomach tumors of the mice were collected, and photos were taken. Animal care and all laboratory procedures were approved by the Animal Ethics Committee of the Inner Mongolia People's Hospital.

### Lentiviral transfection

We purchased viruses (AR, CCRK, shAR or shCCRK) from Shanghai GenePharma Company (Shanghai, China). Transfections were performed according to the manufacturer's instructions.

### Western blot analysis

We used RIPA lysates to cleave cellular proteins and then combined them for use with a BCA protein quantification kit (Thermo Fisher, USA). Protein was isolated from each sample and separated using 10% SDS-PAGE prior to being transferred onto polyvinylidene fluoride (PVDF) membranes. The PVDF membranes were then incubated with 8% milk, followed by incubation with primary antibodies (AR: Abcam, ab9474; CCRK: Abcam, ab227077) and secondary antibodies. The signals were detected by enhanced chemiluminescence.

### Data analysis

AR and CCRK mRNA expression data (GDC TCGA Stomach Cancer) were downloaded from the UCSC Xena database. The clinical data were obtained from the cBioPortal database website. Data analysis of the correlation between AR and CCRK was performed with GraphPad Prism 5.0 (GraphPad Software, USA). We found the genes related to AR in GC tissues from cBioPortal. ChIP-Seq data for AR were downloaded from GEO (GSM2219852). We used the intersection of the top fifty percent of AR-related genes and the top fifteen percent of putative AR targets to analyze their functional categories with the DAVID website. We used X-tile software to find AR and CCRK cut-off values (2.8, 5.4) to maximize the Kaplan-Meier survival curve difference. A student's t-test was used for comparisons between groups. Pearson's correlation analysis was performed to analyze the correlation between AR and CCRK mRNA expression. Kaplan-Meier survival curves and log-rank tests were used to evaluate the overall survival (OS) corresponding to AR and CCRK mRNA expression. When the P value was less than 0.05, the difference was considered statistically significant.

## Results

### AR expression is upregulated in GC

**Figure 1:**
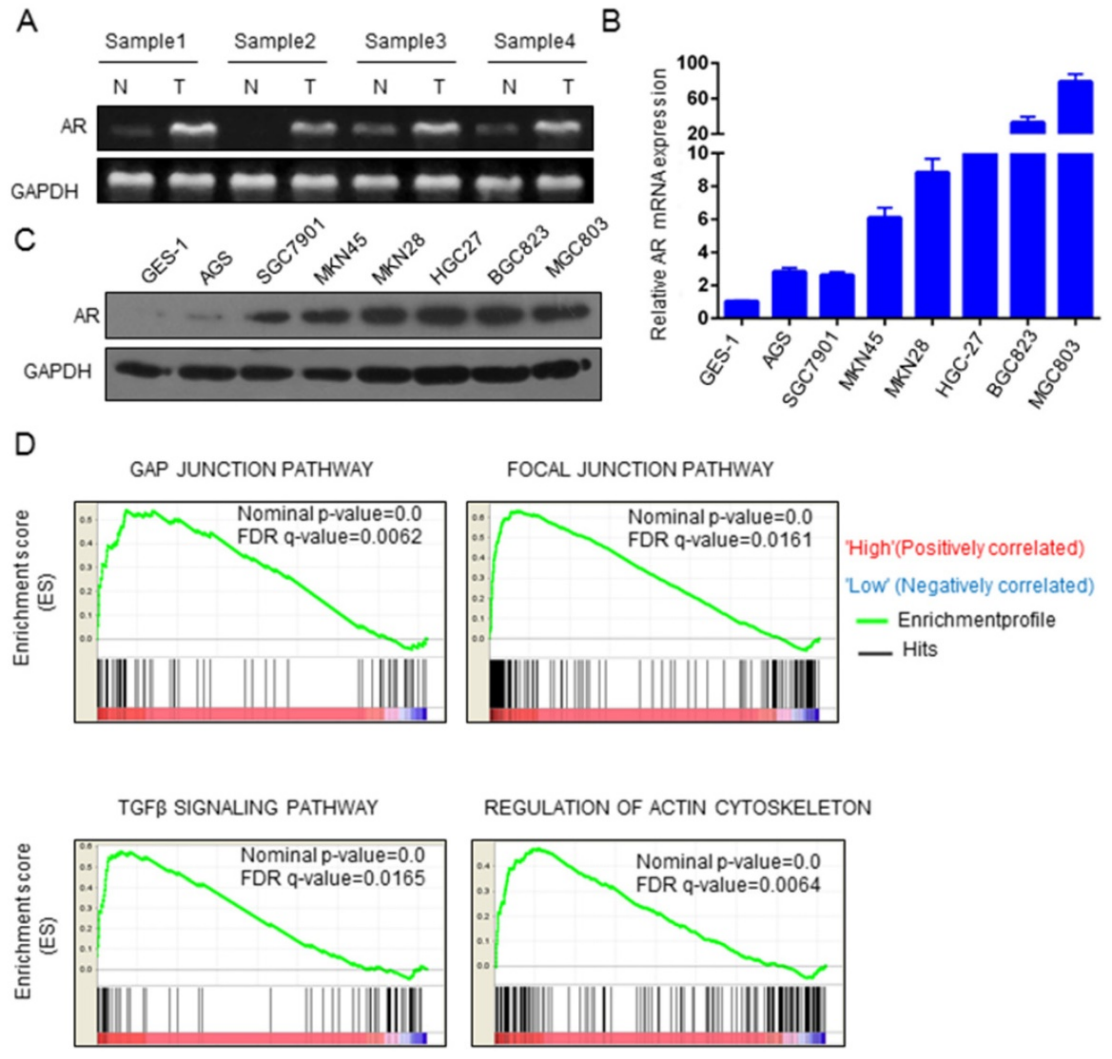
** AR expression was significantly upregulated in GC.** A) Expression of AR mRNA in GC and paracancer tissues. B) Expression of AR mRNA in GC cell lines. C) AR protein levels in GC cell lines. D) The GSEA results of the four pathways are shown, with NES values of 2.21, 1.98, 1.94 and 2.12, respectively, in GC patients.

It has been reported that the expression of AR is higher in GC tissues than in adjacent tissues 16. In our study, RT-PCR was used to verify the expression level of AR mRNA, and in 4 paired clinical GC samples, the expression of AR was, indeed, higher in cancer tissues than in adjacent tissues (Figure 1A). We also found that AR was overexpressed in GC cell lines (6/7) compared to AR expression in gastric immortalized GES-1 cells (Figure 1B). Similar results were found for AR protein levels (Figure 1C). We used GSEA to explore the role of AR in GC progression. The analysis revealed that AR was enriched in cancer-related pathways. AR may be associated with the focal adhesion pathway, the gap junction pathway, the ECM receptor interaction pathway, regulation of the actin cytoskeleton pathway and the TGF beta signaling pathway (Figure 1D). These signaling pathways are involved mainly in cancer metastasis and proliferation.

### AR promotes the migration, invasion and cloning formation ability of GC cells

**Figure 2:**
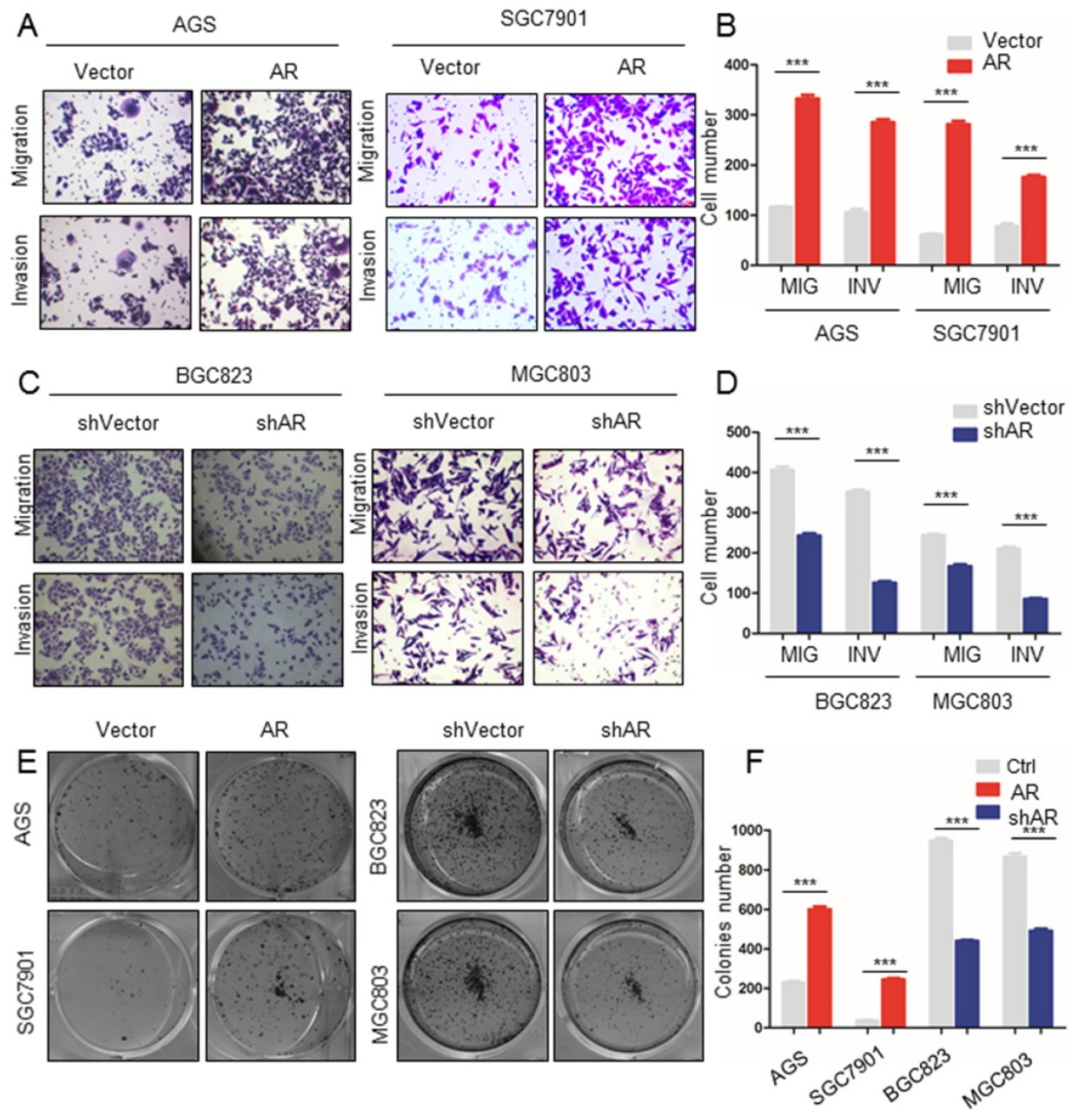
** Upregulation of AR expression promoted GC cell migration, invasion and colony formation.** A) The results of the Matrigel invasion and transwell migration assays showed that the AGS and SGC7901 cell lines overexpressing AR had significantly higher cell invasion and migration than the control groups. B) Transmitted cell numbers in the transwell assay in (A), student's t-test was used for comparisons between groups, p value<0.001. C) The results of the Matrigel invasion and transwell migration assays showed that the number of invaded and migrated cells was significantly lower in the BGC823 and MGC803 cell lines with AR gene knockdown than in the control cells. D) Transmitted cell numbers in the Matrigel invasion and transwell migration assays in (C), student's t-test was used for comparisons between groups, p value <0.001. E) The colony formation assay showed that the AGS and SGC7901 cell lines with high AR expression had significantly higher colony numbers than the control cells. When AR was knocked down in BGC823 and MGC803 cells, the opposite trend was observed. F) Relative colonies numbers in (E), student's t-test was used for comparisons between groups. P value< 0.001.

Based on the GSEA results, we explored whether AR affected the migration and invasion of GC cell lines. Transwell experiments showed that when AR was overexpressed in AGS cells, the migration ability of the cells was increased (Figure 2A, B). A Matrigel transwell assay showed that the invasive ability was significantly increased in AGS cells with overexpressed AR (Figure 2A, B). Similar results were found in SGC7901 cells (Figure 2A, B). Then, we constructed BGC823 and MGC803 cell lines transfected with control vector or shAR. The migration and invasive abilities of the cells were decreased in MGC823 cells with shAR (P value < 0.001, Figure 2C, D). The above results show that AR promoted the migration and invasion of GC cells. We then used the cloning formation assay in different GC cells to show that the cloning ability of the cells was enhanced when AR was overexpressed, and the opposite result was obtained when AR was knocked down (P value < 0.001, Figure 2E, F). The above results validate the hypothesis that AR promotes GC progression by inducing migration, invasion, and proliferation.

### AR upregulates the expression of CCRK as a transcription factor

**Figure 3:**
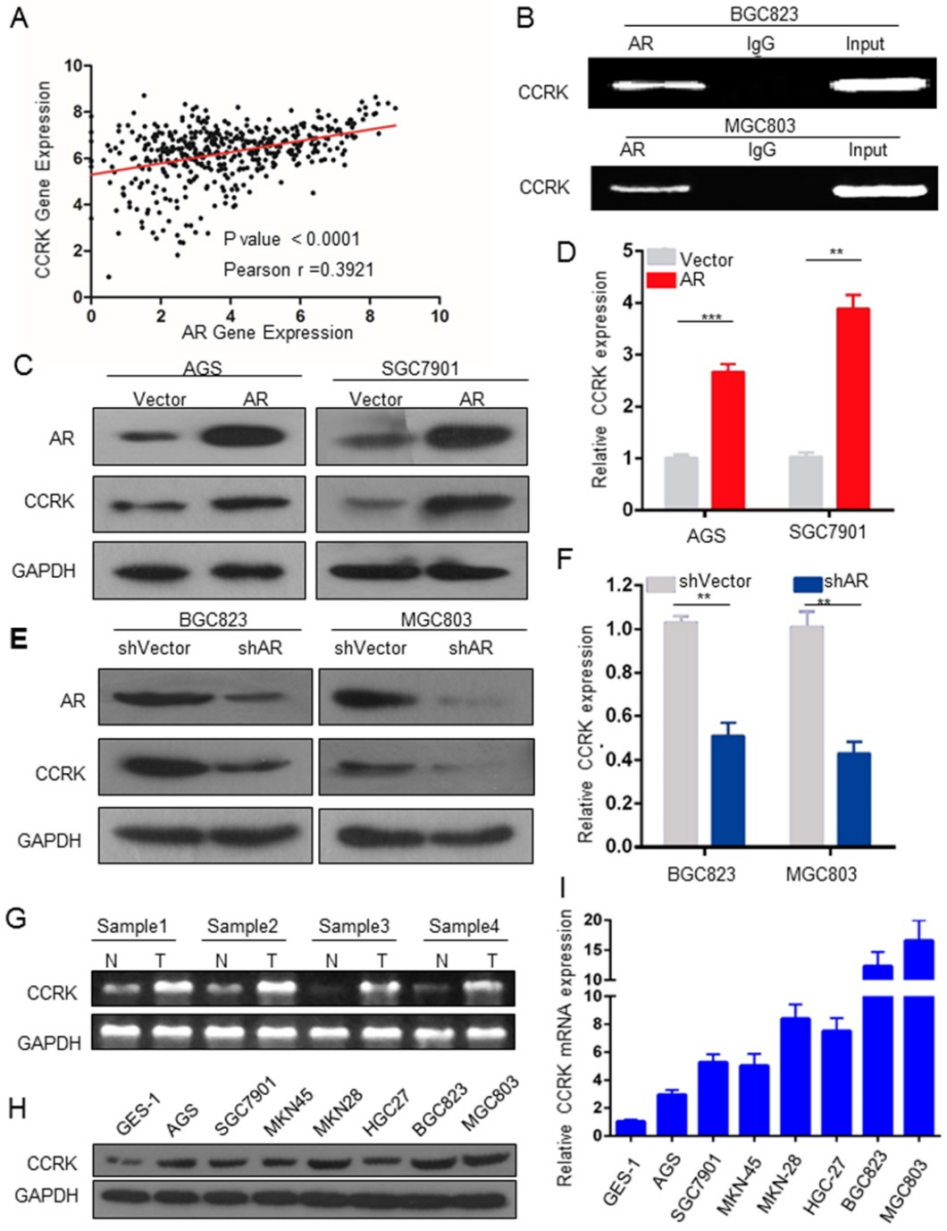
** AR transcriptional regulation promoted CCRK expression.** A) Analysis of the correlation between AR and CCRK expression in GC samples from the TCGA database (r = 0.39, n = 591, P < 0.0001). B) ChIP analysis using an anti-AR antibody. The products were PCR amplified using promoter regions of the CCRK primer. For the control samples, an IgG antibody was added during ChIP. C) D) The ectopic expression efficiency of AR in AGS and SGC7901 cells increased the protein and mRNA expression of CCRK,student's t-test was used for comparisons between groups. E) F) Knockdown of AR in BGC823 and MGC803 cells could decrease the protein and mRNA expression of CCRK. G) CCRK mRNA expression in GC and adjacent tissues,student's t-test was used for comparisons between groups. H) CCRK protein expression in GC cell lines. I) mRNA expression of CCRK in GC cell lines.

We next aimed to explore the underlying mechanisms of how AR promotes GC. We used the intersection of AR-related genes and putative AR targets to analyze their functional categories in DAVID. We focused on 22 genes of cancer-related cell cycle functional categories (Table 1) and analyzed the literature on these 22 genes. Feng H. *et al.* found that in HCC, AR could promote the expression of CCRK through transcriptional regulation 17. Therefore, we wondered whether AR was related to CCRK in GC. We found that the expression levels of AR and CCRK were positively correlated (Figure 3A). To determine whether AR regulates the expression of CCRK through transcription in GC, we conducted ChIP-PCR experiments and found that AR bound specifically to the CCRK promoter in the BGC823 and MGC803 cell lines (Figure 3B). Then, RT-PCR and Western blot analysis were performed in the AGS and SGC7901 cell lines, and the results showed that AR overexpression could promote CCRK expression (Figure 3C, D). Moreover, the mRNA and protein levels of CCRK were decreased when AR was knocked down (Figure 3E, F). We also analyzed 4 pairs of clinical GC samples and found that the mRNA level of CCRK was higher in cancer tissues than in adjacent normal tissues (Figure 3G). In addition, RT-PCR and Western blot analysis showed that the mRNA and protein levels of CCRK were higher in GC cell lines than in gastric immortal cells (Figure 3H, I). Based on the above studies, we speculate that AR promotes the progression of GC through the transcriptional regulation of CCRK expression.

### CCRK promotes the migration, invasion and cloning formation ability of GC cells

**Figure 4:**
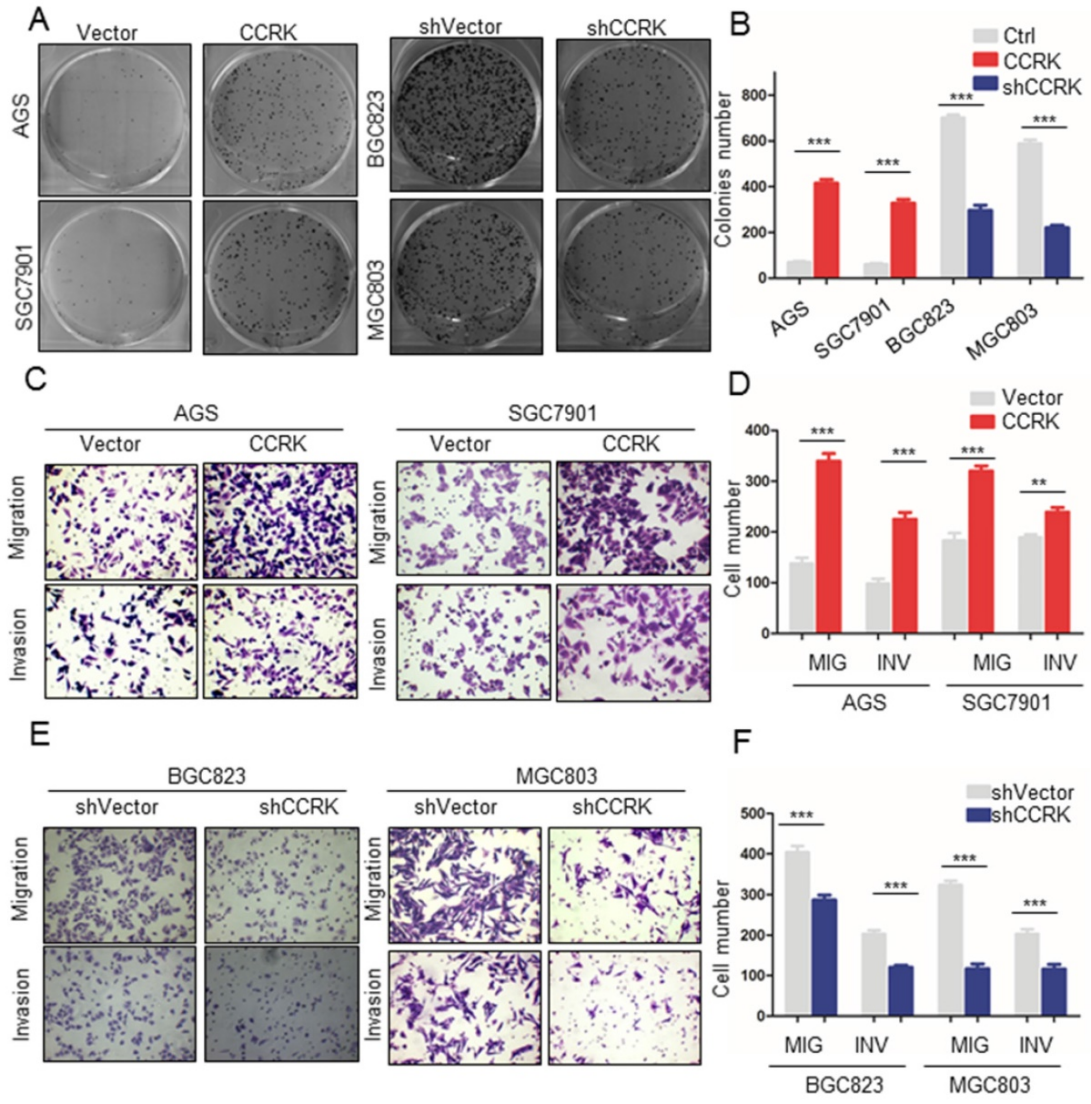
** Overexpression of CCRK promoted migration, invasion and colony formation in GC cells.** A) Colony formation assays showed that the number of colonies was significantly higher for AGS and SGC7901 cells with high CCRK expression than for control cells. When AR was knocked down in BGC823 and MGC803 cells, the opposite trend was observed. B) Comparison of the colony numbers in (A), student's t-test was used for comparisons between groups, P value <0.001. C) The results of the Matrigel invasion and transwell migration assays showed that the AGS and SGC7901 cell lines with high CCRK expression had significantly higher numbers of invaded or migrated cells than the control group. D) Transmitted cell numbers in the transwell assay in (C), student's t-test was used for comparisons between groups, p value<0.001. E) The results of the Matrigel invasion and transwell migration assays showed that the number of cells invaded or migrated was significantly lower for BGC823 and MGC803 cells with CCRK gene knockdown than for control cells. F) Transmitted cell numbers in the Matrigel invasion and transwell migration assays in (E), student's t-test was used for comparisons between groups, P value <0.001.

Since AR was found to regulate the expression of CCRK transcriptionally, we wondered whether CCRK might also affect the progression of GC. Therefore, we conducted a series of functional experiments on CCRK in GC cell lines. Cloning formation experiments showed that in the AGS and GES-1 cell lines, CCRK overexpression could enhance the clonal formation ability of the cells (P value <0.001, Figure 4A, B). When CCRK was overexpressed in the AGS and SGC7901 cell lines, the migration and invasion abilities of the cells were increased significantly (Figure 4C, D). Subsequently, CCRK was knocked down in the BGC823 and MGC803 cell lines. The results showed that the cloning formation ability of the cells was decreased after CCRK knockdown (Figure 4A, B), and the migration and invasion abilities were also decreased (Figure 4E, F). These results show that CCRK promotes the cloning formation, migration and invasion of GC cells *in vitro*.

### CCRK promotes the growth of xenografted tumors in nude mice

**Figure 5:**
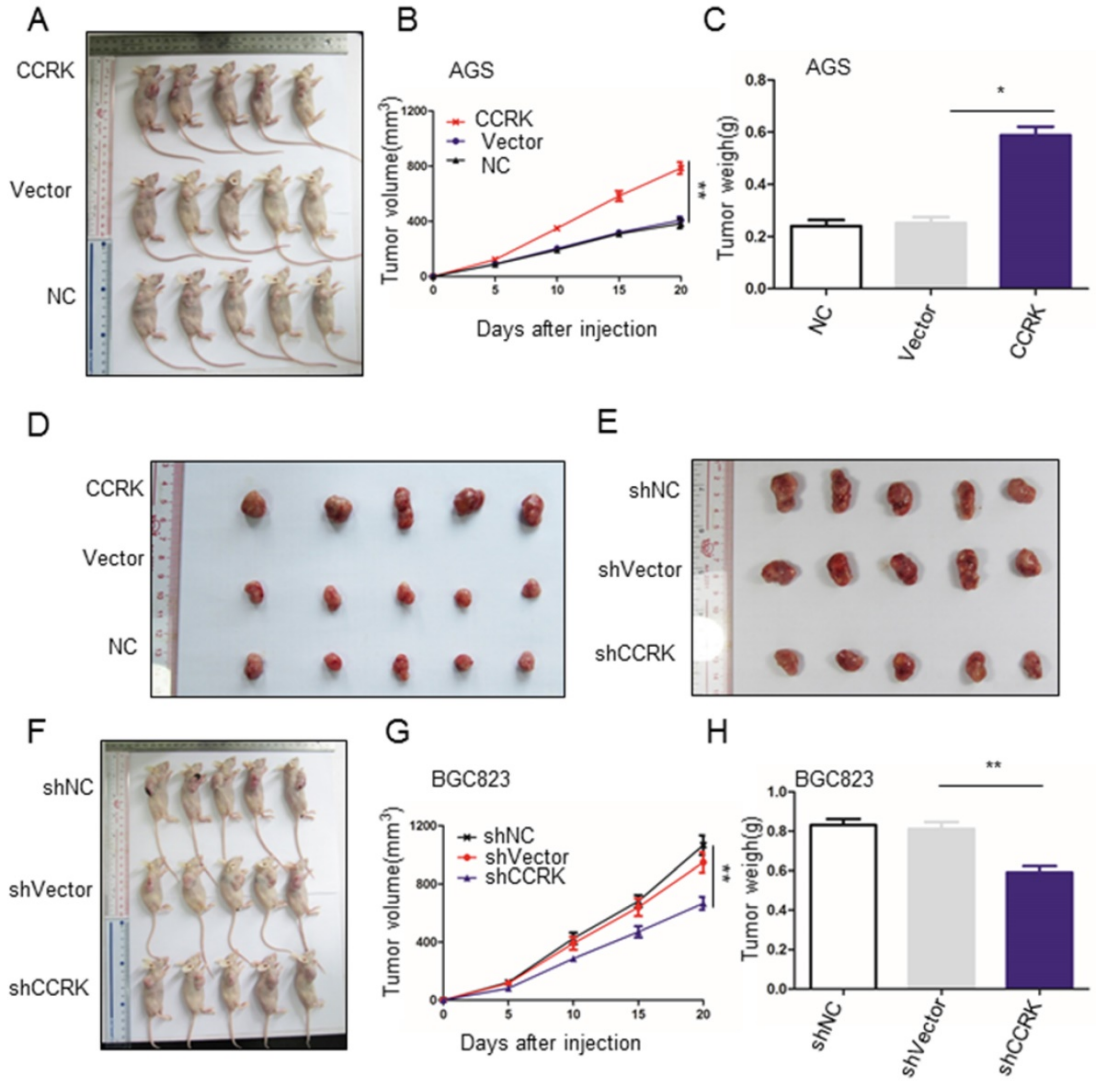
** CCRK affected the growth of xenograft tumors in nude mice.** A) B) CCRK overexpression promoted the growth of transplanted tumors in nude mice. At the end of the experiment, xenograft tumors were collected from the nude mice that were injected subcutaneously with AGS cells expressing CCRK or carrying either a control vector or no vector. C) Tumor volume growth curve for (A). D) Tumor weights from (A) compared by Student's t-test. E) F) ShCCRK reduced transplanted tumor growth in nude mice. At the end of the experiment, xenograft tumors were collected from the nude mice that were injected subcutaneously with BGC823 cells with CCRK knockdown and with or without a control vector. G) Tumor volume growth curve for (F). H) Tumor weights from (F) compared by Student's t-test.

We further investigated whether CCRK displayed the function of an oncogene *in vivo*, and the results showed that CCRK overexpression significantly increased the volume and weight of xenograft tumors (Figure 5A-D). In addition, CCRK knockdown xenograft tumors showed a reduced growth rate (Figure 5E-H). These data suggest that CCRK acts as a tumor oncogene in xenograft mouse models.

### Overexpression of AR increases the level of p-GSK3β, p-β-catenin and EGFR

**Figure 6:**
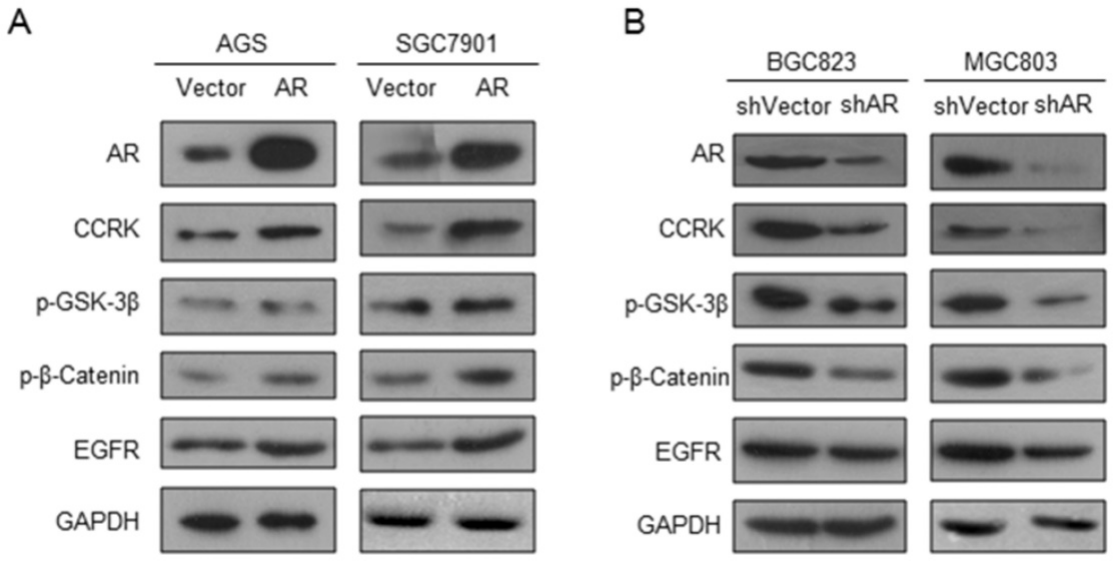
** AR promoted GC by modulating p-GSK-3β, p-β-catenin and EGFR.** The effects of the A) ectopic expression or B) knockdown of AR on CCRK, p-GSK-3β, p-β-catenin and EGFR were analyzed by Western blot assay. GAPDH protein levels were used as an internal control.

We examined the mechanism of how AR promoted GC. Feng H. *et al.* found that in male hepatocellular carcinoma, androgen-dependent AR activation induced CCRK expression, which led to the phosphorylation of GSK3β and β-catenin. This activation stimulated the expression of β-catenin-TCF4-dependent target genes such as cyclin D1, epidermal growth factor receptor (EGFR) and glutamine synthase (GS) 17. Therefore, we wondered whether there are similar mechanisms in GC. In our experiments, we found that when we upregulated AR expression, the CCRK levels were increased in GC cell lines, as well as the levels of p-GSK3β, p-β-catenin and EGFR (Figure 6A). Moreover, when we knocked down AR, CCRK expression was reduced, and the level of p-GSK3β, p-β-catenin and EGFR were reduced (Figure 6B). Therefore, we believe that AR affects the migration, invasion and cloning formation in GC cells by upregulating CCRK, p-GSK3β, p-β-catenin and EGFR.

### AR-CCRK has the potential to be a prognostic indicator for GC patients

**Figure 7:**
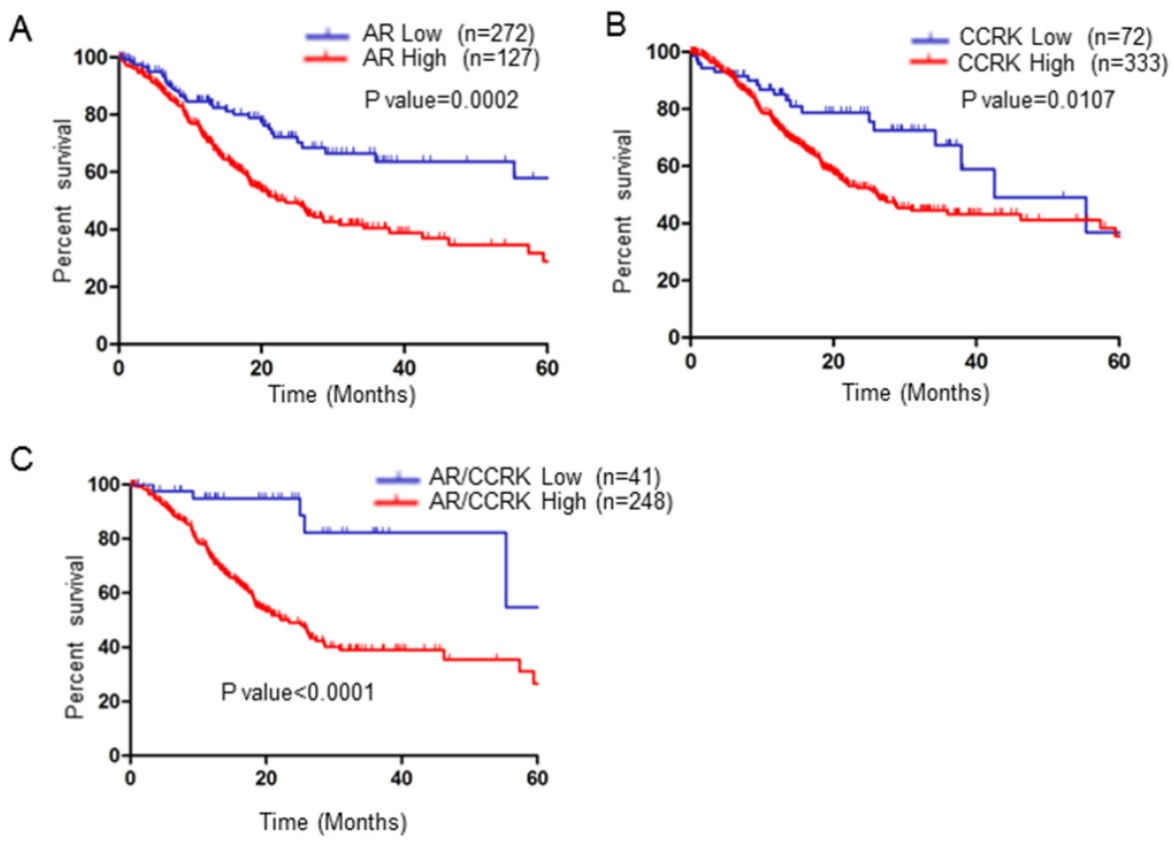
** AR and CCRK were associated with poor prognosis in patients with GC.** Log-rank tests were used to evaluate the overall survival (OS) A) The overall survival (OS) data of GC patients from TCGA with low or high AR expression were analyzed by Kaplan-Meier survival analysis. B) The OS data of GC patients from TCGA with low or high CCRK expression were analyzed by Kaplan-Meier survival analysis. C) The OS data of GC patients from TCGA with low or high AR/CCRK expression were analyzed by Kaplan-Meier survival analysis.

Finally, we investigated the clinical application prospects of AR and CCRK. We analyzed the prognostic ability of AR and CCRK in GC patients with TCGA data. As shown by the Kaplan-Meier survival curve, GC patients with lower AR mRNA expression had significant survival advantages over those with higher AR expression (P value = 0.0002, Figure 7A). CCRK was analyzed at the same time, and GC patients with low CCRK mRNA expression also had survival advantages over patients who had high CCRK expression (P value=0.0107, Figure 7B). Surprisingly, the group of patients with low expression of both AR and CCRK had a more obvious survival advantage than the group of patients with high expression of both AR and CCRK (P value < 0.0001, Figure 7C). This survival analysis indicates that AR combined with CCRK has the potential to be a prognostic indicator for GC patients.

## Discussion

Androgen receptor (AR) is well-known as a target of prostate cancer which is a malignancy in the male reproductive system 18. AR has also been reported to play an important role in the male-dominant nature of HCC 19. In this study, we found that AR expression was higher in GC tissues than in adjacent tissues. Upregulation of AR expression promoted colony formation, cell invasion and migration in GC cells. In contrast, AR knockdown resulted in a decrease in cell colony formation ability, invasion and migration in GC cells. These results validated the carcinogenic role of AR in the progression of GC. Since AR showed carcinogenic effects in our study, some AR antagonists may also be used to block the progression of GC. Bicalutamide and enzalutamide are AR inhibitors that restore endocrine therapy sensitivity when used in anti-hormone therapy for breast cancer 20. The successful application of enzalutamide played an important role in the development of new AR antagonists. ARN-509, a next-generation antiandrogen, could improve patients' pharmacological tolerance characteristics and has better application potential 21. Research on the inhibition of ligand-independent AR pathways, such as through AR mutants or variants (vs), has been ongoing. For example, in castration-resistant prostate cancer (CRPC), galeterone is an interesting candidate that promotes the degradation of AR and AR vs 22. It has been reported in the literature that in prostate cancer, ROR-γ could promote the expression of AR by transcription, and the application of an ROR-γ antagonist had an effect on the castration of prostate cancer. The ROR-γ antagonist could inhibit AR-positive tumor cell proliferation by reducing AR or AR-V7 (AR vs) 23. These AR antagonists may suppress GC progression. Mitchell S. H. *et al.* found that AR gene amplification was estimated to be present in approximately one-third of patients with recurrent cancer 24. This finding also suggested that AR may be used as a prognostic predictor for GC. We analyzed the TCGA database and found that patients with high AR expression who had a worse prognosis than those with low AR expression. This result was consistent with the role of AR in promoting cancer. Functional experiments also suggested that high AR expression promoted the migration and invasion of GC, thus leading to the poor patient prognosis.

Our data demonstrated that AR could promote CCRK expression in GC through transcriptional regulation. Transwell experiments showed that CCRK increased invasion and metastasis in GC cells. Our results also showed that the high expression of CCRK increased the ability of cells to clone; subcutaneous tumorigenesis experiments in nude mice showed that CCRK knockdown reduced the tumor volume, but CCRK overexpression increased the tumor volume. Our experimental results indicate that CCRK is an oncogene in GC. Therefore, CCRK antagonists may also play a role in the progression of GC. CDK inhibitors have also been used to inhibit the expression of CCRK. Mueller *et al.* tested six commercially available inhibitors (flavonoid piperidol, roscovitine, parboxiklin, sorafenib, minocycline, and ponatinib) and one preqin enzyme development inhibitor (MER151). The authors found moderate inhibitory effects *in vitro* because these compounds were not completely specific for CCRK^25^. Currently, there are no published reports on the development of CCRK-specific inhibitors. One of the main reasons may be the lack of a three-dimensional structure of the CCRK protein. Currently, although there is no specific and effective inhibitor of CCRK, CCRK may be used as an indicator for GC prognosis. Our study suggested that patients with high CCRK expression had a poor prognosis, while patients with low CCRK expression had a good prognosis. In GC, patients with high expression of both AR and CCRK had more significant prognostic differences than those with low expression of both AR and CCRK. The prognosis of low AR and CCRK expression patients was significantly better than that of high AR and CCRK expression patients. Therefore, AR and CRRK have potential as prognostic indicators of GC.

In summary, we show that the expression of CCRK is increased by AR in GC. Low expression levels of AR and CCRK are related to a better prognosis in GC patients. AR and CCRK can promote the development of GC, suggesting that we should investigate the effects of AR or CCRK inhibitors. In addition, these molecules are strong candidates to be prognostic indicators of GC.

## Figures and Tables

**Table 1:** Functional categories for the intersection of AR-related genes and putative AR targets

Term	P Value	Genes
Cell cycle	0.047	CDC7, EID1, MKI67, PIM1, AURKA, CD2AP, NCAPH, MCM7, TSPYL2, RAB11FIP3, HEPACAM, ZWINT, USP39, MTBP, NEK9, PELO, BRINP3, MAP9, CDK20, NSUN2, BUB3, CDK14
Neurogenesis	0.019	MEF2A, NTNG2, SLIT2, BZW2, PRKD1, EPHA5, SEMA6A, EPHA7, SEMA6D, NEDD4, CHN1, ZC3H12A, EFNA5, ACTL6A
Mental retardation	0.018	PGAP2, TUSC3, ZBTB16, DOCK8, MAPK10, FOXP1, NANS, ZDHHC9, AUTS2, IL1RAPL1, RBM28, NSUN2, HPD
Steroid metabolism	0.009	CYB5R3, CYP46A1, HMGCR, HSD11B1, PRKAA2, ABCA1, DHCR24
Tight junction	0.009	CLDN7, TJP1, MPDZ, CLDN10, EPB41L4B, CLDN12, LIN7A
Cholesterol metabolism	0.008	CYB5R3, CYP46A1, HMGCR, PRKAA2, ABCA1, DHCR24

## Abbreviations

GC: gastric cancer
CCRK: cell cycle related kinase
AR: androgen receptor
GSEA: Gene enrichment analysis
ES: enrichment score
NES: normalized enrichment score
FDR: false discovery rate
TCGA: the cancer genome atlas
ChIP: chromatin immunoprecipitation
PVDF: polyvinylidene fluoride
EGFR: epidermal growth factor receptor
GS: glutamine synthase
vs: variants
CRPC: castration resistant prostate cancer
OS: overall survival
