# Androgen Receptor Promotes Gastric Carcinogenesis via Upregulating Cell Cycle-Related Kinase Expression: Erratum

**DOI:** 10.7150/jca.117489

**Published:** 2025-06-15

**Authors:** Ren Wang, Xiao-yi Xu, Hong Zhu, Xiong Liang, Xue Li, Ming-xu Jia, Qing-hua Wang, Hui-yun Wang, Xiao-xing Li, Gui-jun Zhao

**Affiliations:** 1 State Key Laboratory of Oncology in South China, Collaborative Innovation Center for Cancer Medicine, Sun Yat-Sen University Cancer Center, Guangzhou, Guangdong, China; 2 Endoscopy Center, Inner Mongolia key laboratory of endoscopic digestive diseases, Inner Mongolia People's Hospital, Hohhot, Inner Mongolia Autonomous Region, China

In our published paper, we regret to note that when selecting representative images from a large volume of data, the images displayed in Figures 2, 4, 5, and 6 were inadvertently misused. Below are the corrected versions of these figures. The authors confirm that the corrections in this erratum do not change the overall conclusions of the study. We sincerely apologize for any errors and any inconvenience caused.

## Figures and Tables

**Fig 2:**
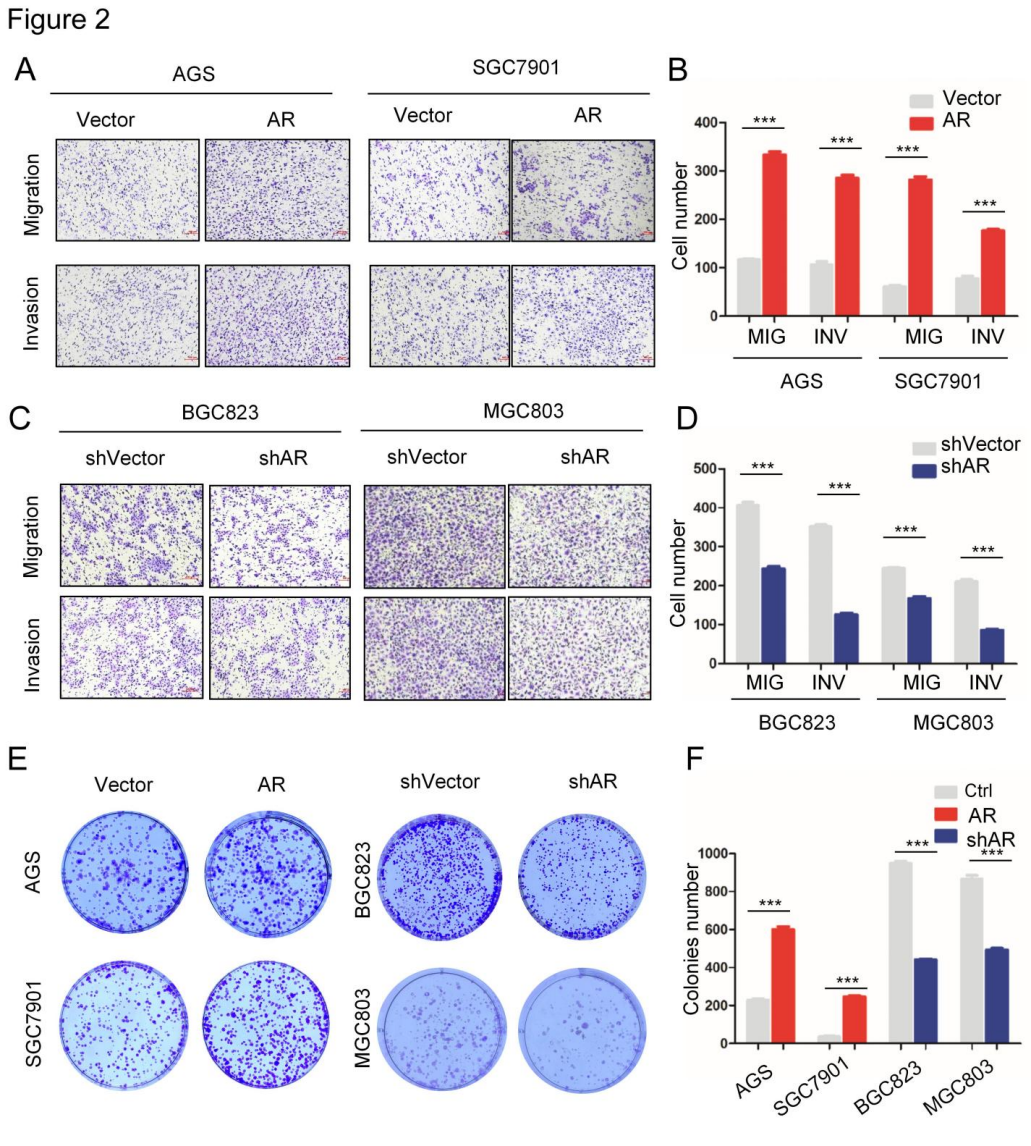
Corrected image as shown

**Fig 4:**
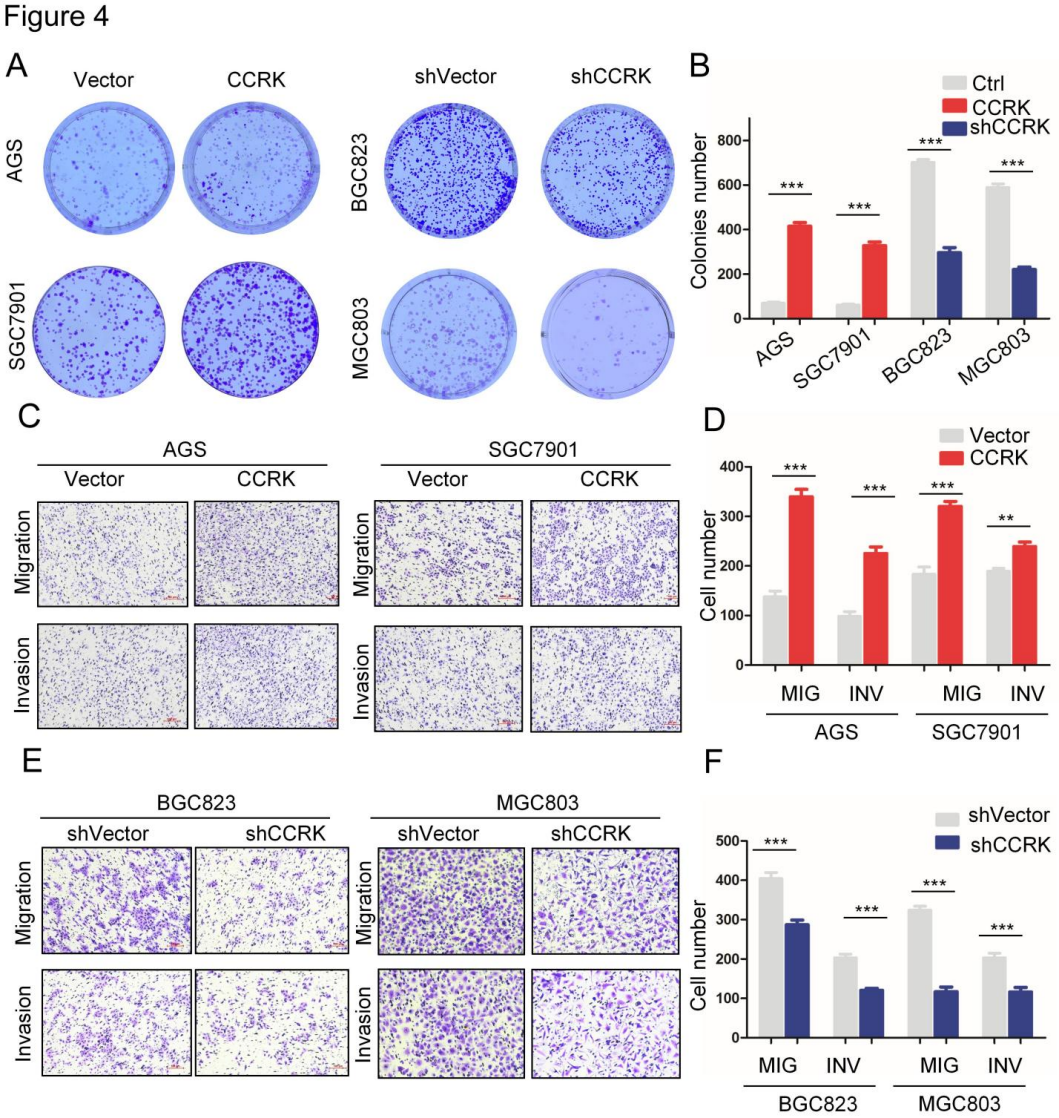
Corrected image as shown

**Fig 5:**
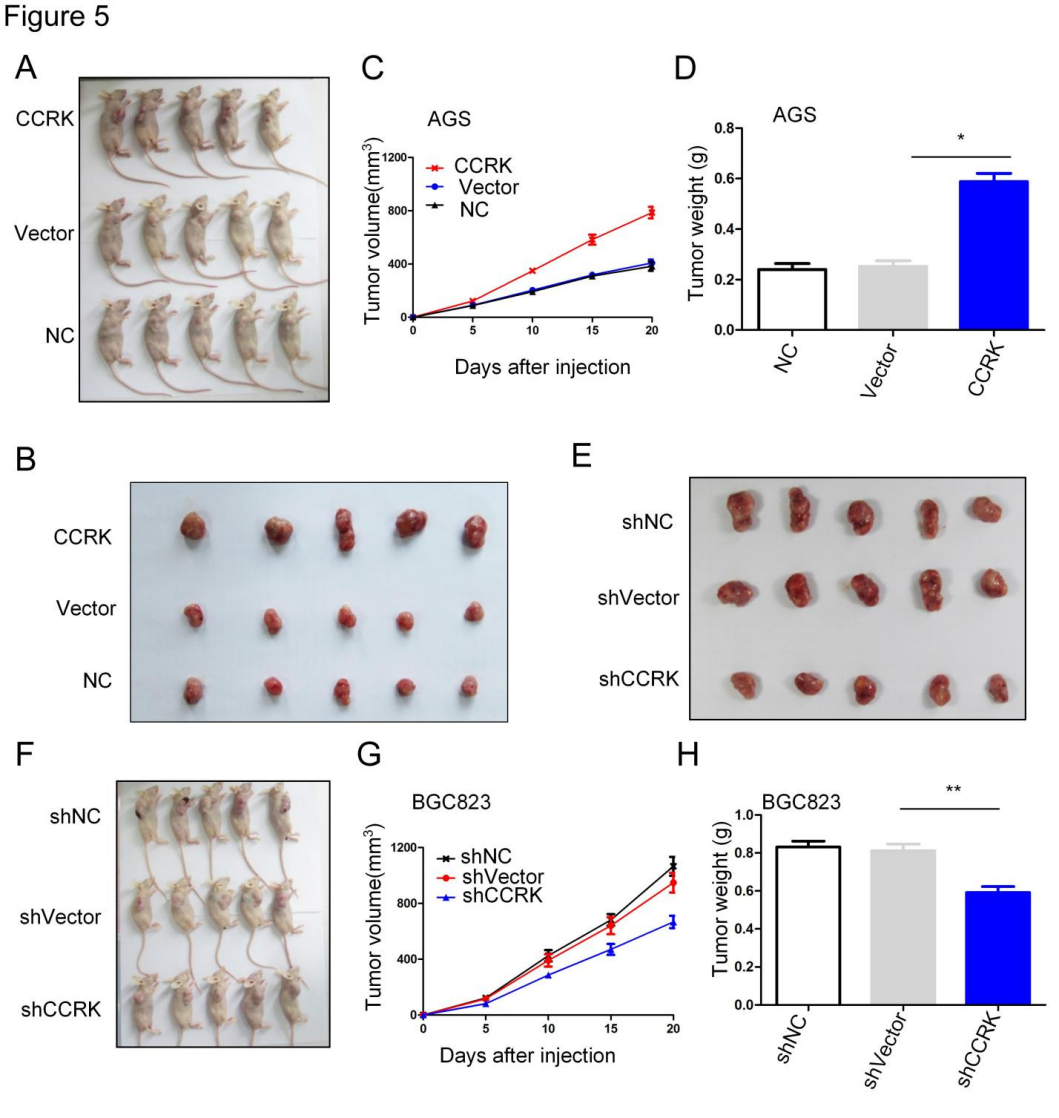
Corrected image as shown

**Fig 6:**
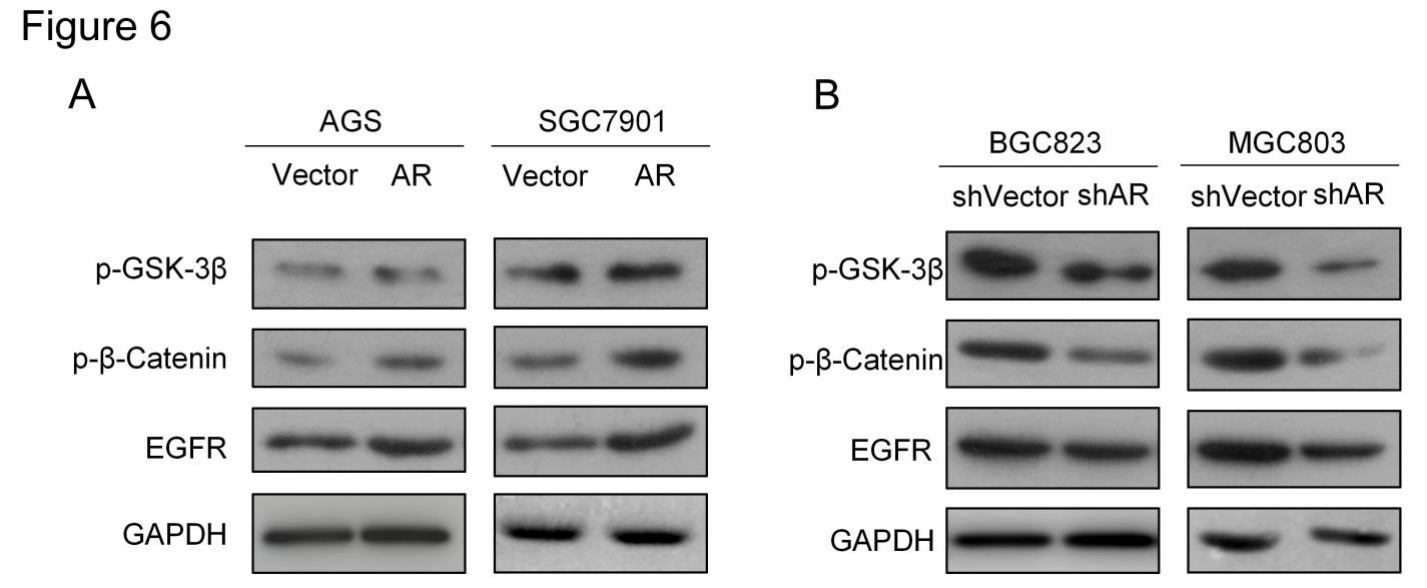
Corrected image as shown

